# A Novel Hypomorphic *STAT3* Gene Variant in a 7-year-old Male with Hyper-IgE Syndrome

**DOI:** 10.1007/s10875-025-01942-7

**Published:** 2025-10-20

**Authors:** Tomoko Higashigawa, Yukiko Ikeyama, Kosuke Ashihara, Takaki Asano, Satoshi Okada, Yuki Miwa, Katsumi Sugiura, Hidenori Ohnishi

**Affiliations:** 1 Department of Pediatrics, Matsusaka Chuo General Hospital, 102 Kawaimachi, Matsusaka, 515-8566 Mie Japan; 2 Department of Pediatrics, Hiroshima University Graduate School of Medicine and Health Sciences, Hiroshima, Hiroshima Japan; 3 Department of Pediatrics, Gifu University School of Medicine, Gifu, Gifu Japan; 4 Department of Genetics and Cell Biology, Research Institute for Radiation Biology and Medicine, Hiroshima University, Hiroshima, Japan

**Keywords:** Hyper-IgE syndrome, Hypomorphic, Dominant-negative, Primary immunodeficiency, STAT3

## To the Editor

Hyper-IgE syndrome (HIES) has been proposed as an immunodeficiency syndrome showing the classic triad of high serum IgE level, recurrent cold abscesses of the skin caused by *Staphylococcus aureus*, and recurrent pneumonia complicated with lung cysts. Several genetic abnormalities are associated with the HIES phenotype, with the main genetic cause being dominant-negative mutations in the signal transduction and activator of transcription 3 (*STAT3*) gene (*STAT3*–HIES) [1]. There are several genetic hotspots for *STAT3* mutations (R382W, R382Q, F384L, V463del, V637M, etc.) [2], accounting for about two-thirds of the total, but > 80 mutations have been reported [3, 4]. In this report, we report a case of HIES with a novel genetic variant diagnosed based on repeated skin infections and family history.

## Case Description

The patient was a 7-year-old male with a chief complaint of an abscess in the groin. Two months before admission, he had been undergoing treatment for recurrent numerous furuncles on the head, upper eyelids, trunk, and extremities. During treatment, he developed abscess formation in the inguinal lymph node and was admitted to our hospital. On admission, he had multiple 5-mm furuncles on his face, scalp, auricle, extremities, and trunk. Some had already self-destructed or crusted over. The 4-cm abscess in the groin was soft, tender, and mildly erythematous.

He had been hospitalized at the age of 5 for an abscess in the lower limb caused by methicillin-resistant *Staphylococcus aureus* (MRSA) cellulitis. No obvious allergy was noted, but he had dry skin and hypersensitivity to insect bites. He had multiple dental caries but no history of bone fractures or pneumonia. The following blood examination results were obtained: C-reactive protein: 0.25 mg/dL and leukocyte count: 5900/µL (eosinophils: 4.6%, bacillary nuclei: 0.0%, segmental nuclei: 51.8%, lymphocytes: 34.3%, and monocytes: 9.1%). These results indicate a poor inflammatory response compared to his clinical symptoms.

MRSA was detected in a puncture culture of the abscess. After 6 days of anti-MRSA treatment, the patient was discharged and the treatment was switched to oral trimethoprim/sulfamethoxazole (TMP/SMX). The inguinal abscess healed well, and the TMP/SMX treatment was completed. One month later, he was readmitted to the hospital with upper-arm and thigh cellulitis. As found earlier, physical examination revealed mixed-stage multiple furuncles on the extremities and trunk. Blood testing results also showed no significant abnormality other than a markedly elevated serum IgE level. Although the cellulitis quickly improved under anti-MRSA therapy, new furuncles continued to appear throughout his body, suggesting that antibiotics were not effective in controlling the appearance of furuncles. However, due to a history of repeated skin bacterial infections, an immunodeficiency was suspected, and he started on TMP/SMX prophylaxis.

Immune evaluation revealed negative antinuclear antibodies, normal complement titers, immunoglobulin (Ig)G/A/M and IgG2, and a normal antibody response to vaccination. Neutrophil count, neutrophil bactericidal/phagocytic activity, and lipopolysaccharide responsiveness test results were all normal. However, his nonspecific serum IgE level was markedly elevated at 6,680 IU/mL. Peripheral lymphocyte surface marker analysis showed no abnormalities in T cells, B cells, or the CD4/8 ratio. Notably, Th17 cells were detected but were relatively low (~ 0.25%) [5, 6].

His National Institutes of Health (NIH) score for HIES was 19, below the typical cutoff [7]; the absence of Th17 deficiency or a history of fractures or pneumonia also did not suggest typical HIES. Further family history interviews revealed that the patient’s father had a history of multiple skin infections when he was < 10 years old. These infections were similar to those of his son; however, they had disappeared in adulthood, leaving only chronic otitis externa at present. Additionally, the father had been hospitalized for pneumonia four times in his late teens, 20 s, and 30 s, and bronchiectasis was diagnosed. He had also experienced five traumatic fractures.

**Fig. 1:**
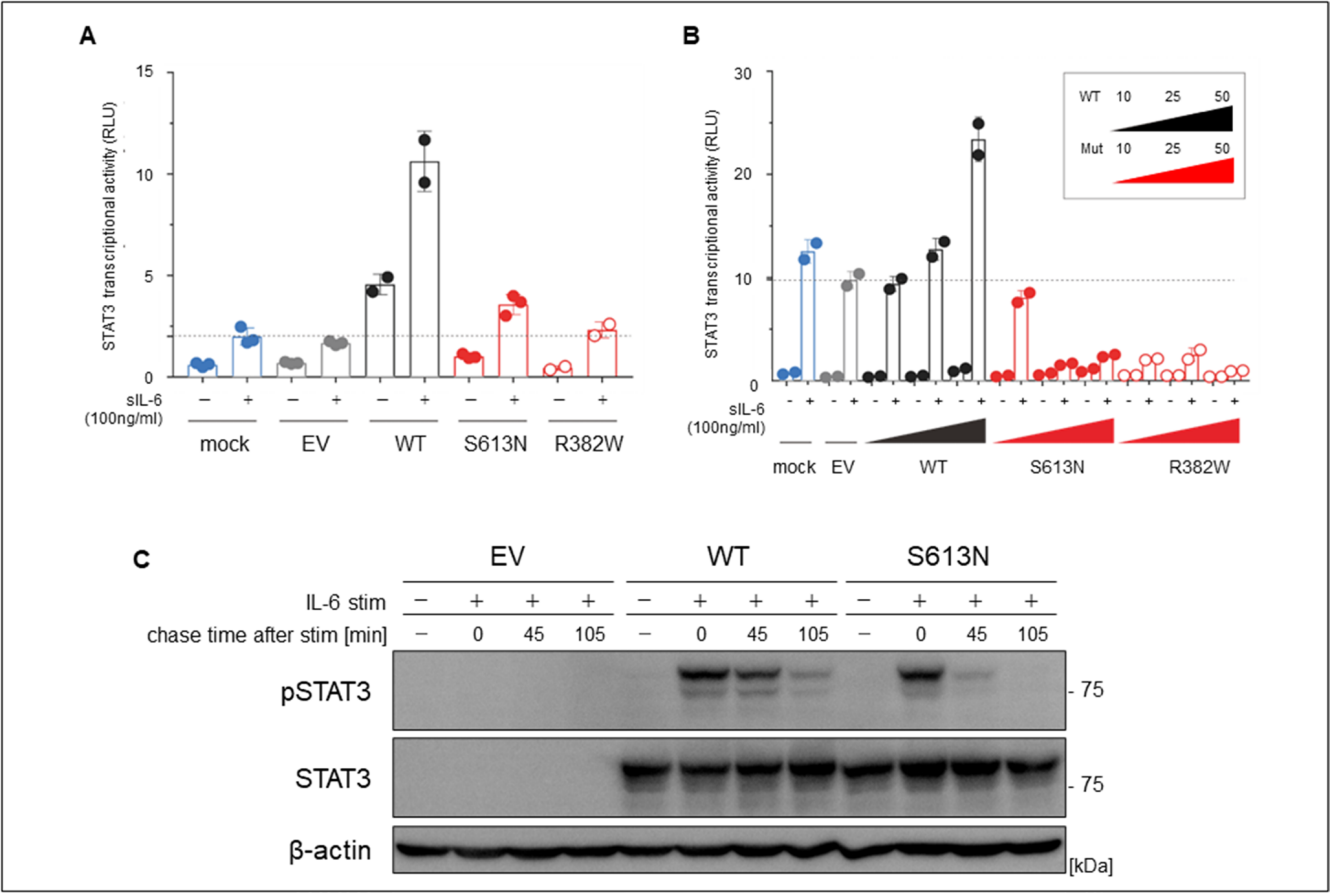
Functional analysis of the S613N variant. (**A**-**C**) Three types of *STAT3* constructs (wild-type [WT] and two mutants [S613N and R382W, known as loss-of-function and dominant-negative]) were transfected into (**A**) *STAT3*^−/−^ A4 cells for luciferase assay and (**B**) HEK293T cells with endogenous *STAT3,* using the pGL4.32 luciferase reporter construct and expression vector for *Renilla* luciferase together with no vector (mock), empty vector (EV), WT, or *STAT3* variants (S613N and R382W). After 24 h, transfected cells were left untreated or treated with 100 ng/mL of sIL-6 for 24 h. The y-axis represents STAT3 transcriptional activity as relative luciferase units. Blue represents mock, gray represents EV, black represents WT, and red represents STAT3 variants. The R382W variants, denoted by red unfilled circles, have previously been reported as loss-of-function and dominant-negative *STAT3* variants, indicating their role in disease control. The black dashed line indicates sIL-6-stimulated EV-transfected cells. These experiments were independently repeated twice. Error bars show the mean ± SEM. (**C**) Immunoblot for STAT3 phosphorylation (pSTAT3) in *STAT3*^−/−^ A4 cells. The cells were transfected with either WT or variant pCMV6-STAT3 plasmids, or EV. After 24 h, transfected cells were left untreated or treated with IL-6 (10 ng/mL ). The S613N variant did not show impaired pSTAT3 protein expression but showed a faster decrease compared with WT

The father’s serum IgE level was ≤ 1,205 IU/mL, with elevated IgE specific for *S. aureus* and *Candida* but no eosinophilia. His Th17 cells were absent (~ 0.03%). Thus, the father’s NIH score was high (43 points), and HIES was strongly suspected [8]. In this patient, the genomic gene sequences of the protein coding regions’ exon and intron boundary regions (intron ≤ 10 bases) of known HIES-causing genes (*STAT3*, *TYK2*, *IL6R*, *ZNF341*, *ERBIN*, *TGFBR1*, *TGFBR2*, *SPINK5*, *PGM3*, *CARD11*, *DOCK8*) were analyzed by targeted next-generation sequencing using the hybrid capture method. The obtained sequences were compared with the publicly available human genome reference sequence (GRCh38/hg38), analyzed for low-frequency base substitutions, short-base deletions and insertions, and variants not listed in the database. The variants with a frequency of ≤ 1% were designated as novel gene mutations. The variant frequency information databases were gnomAD_v3.1, iJGVD_8.3kjpn, CLINSIG, Clinvar_20220723, and HGMD, 2022.2. Accordingly, we identified a novel missense variant in *STAT3* (c.1838G > A, p.Ser613Asn). Ser613 is located in the SH2 domain, which is one of the functional domains of STAT3. This variant was verified by performing the Sanger method for the patient and his parents, which revealed a similar variant only in his father (Supp. Figure 1). A transient expression system was used to investigate the functional consequences of the S613N variant and wild-type (WT) plasmids. Each plasmid was introduced by lipofection into *STAT3*-deficient A4 and 293 T cells. The cells were then stimulated with soluble IL-6 for 24 h. *STAT3* transcriptional activity was lower in the A4 cells expressing S613N *STAT3* than in those expressing the wild-type, indicating that this variant was hypomorphic. The S617N variant exerted a dose-dependent negative effect on wild-type–mediated *STAT3* transcriptional activity in HEK293T cells, suggesting that the S613N variant is dominant-negative (Fig. 1A, B). Transfection into A4 cells with WT or variants of pCMV6-STAT3 plasmids resulted in the S613N variant showing a faster decrease in the expression level of pSTAT3 protein than WT (Fig. 1C). Additionally, we assessed the expression levels of *SOCS3* affected by STAT3 variants (Supp. Figures 2 and 3). In this result, we also observed a relatively lower expression level of SOCS3 with the STAT3 S613N variant in the in vitro experiment.

## Discussion

HIES is characterized by *S. aureus* infections of the lungs and skin caused by two factors: the important role of Th17 cells in defense against *S. aureus* infections and the greater reliance of skin keratinocytes and bronchial epithelial cells on Th17 cells for defense against *S. aureus* infection than those in other tissues [8]. Both the father and son mainly had skin infections and no pneumonia until their early teens. The patient also had no recent history of severe pneumonia, and it is unclear why the same genetic mutation would cause the infection site to change with age.

The *STAT3* functions as a signaling hub for > 40 cytokines, hormones, and growth factors, affecting the immune system as well as connective tissue, cardiovascular system, bone, and tooth formation. Therefore, aging may alter infection susceptibility sites as local immune functions in organs mature or degenerate.

For the clinical diagnosis of HIES, the NIH score based on symptoms and laboratory values has been proposed and is useful for screening. However, the diagnosis is often difficult in childhood because of the lack of these clinical symptoms. In our case, the patient and his father had the same genetic variant, but Th17 cells in the peripheral blood were detected only in the proband. Long-term follow-up of the patient for age-related changes in clinical symptoms and Th17 cell counts is needed to clarify the natural history of HIES.

## Supplementary Information

Below is the link to the electronic supplementary material.

Supplementary figure 1 PNG 144 KB

High Resolution Image (TIF 11.8 MB)

Supplementary figure 2 PNG 29.6 KB

High Resolution Image (TIF 1.13 MB)

Supplementary figure 3 PNG 356 KB

High Resolution Image (TIF 12.4 MB)

Supplementary file4 (DOCX 16.4 KB)

## Data Availability

The datasets generated during and/or analysed during the current study are available from the corresponding author on reasonable request.
