# Supplementary figures and images for: A Novel Hypomorphic STAT3 Gene Variant in a 7-year-old Male with Hyper-IgE Syndrome
Source: J Clin Immunol. 2025 Oct 20;45(1):146. doi: 10.1007/s10875-025-01942-7 (PMC12537600; doi:10.1007/s10875-025-01942-7)

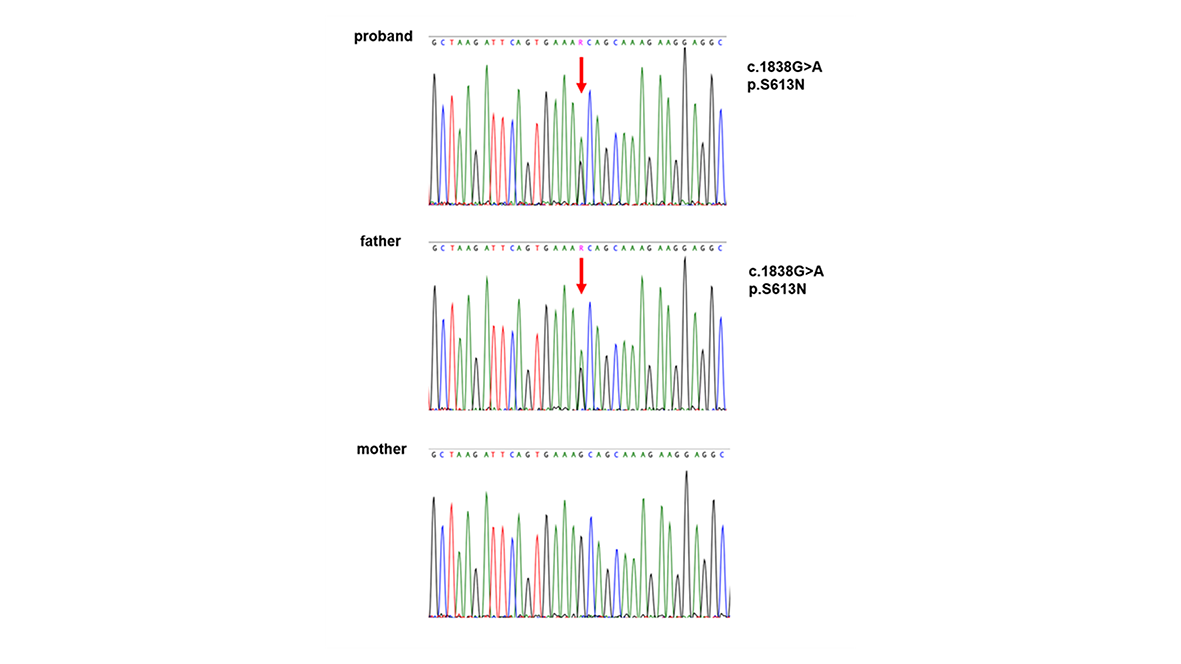

Supplement: Supplementary file 1 — PNG 144 KB [file 10875_2025_1942_Fig2_ESM.png]

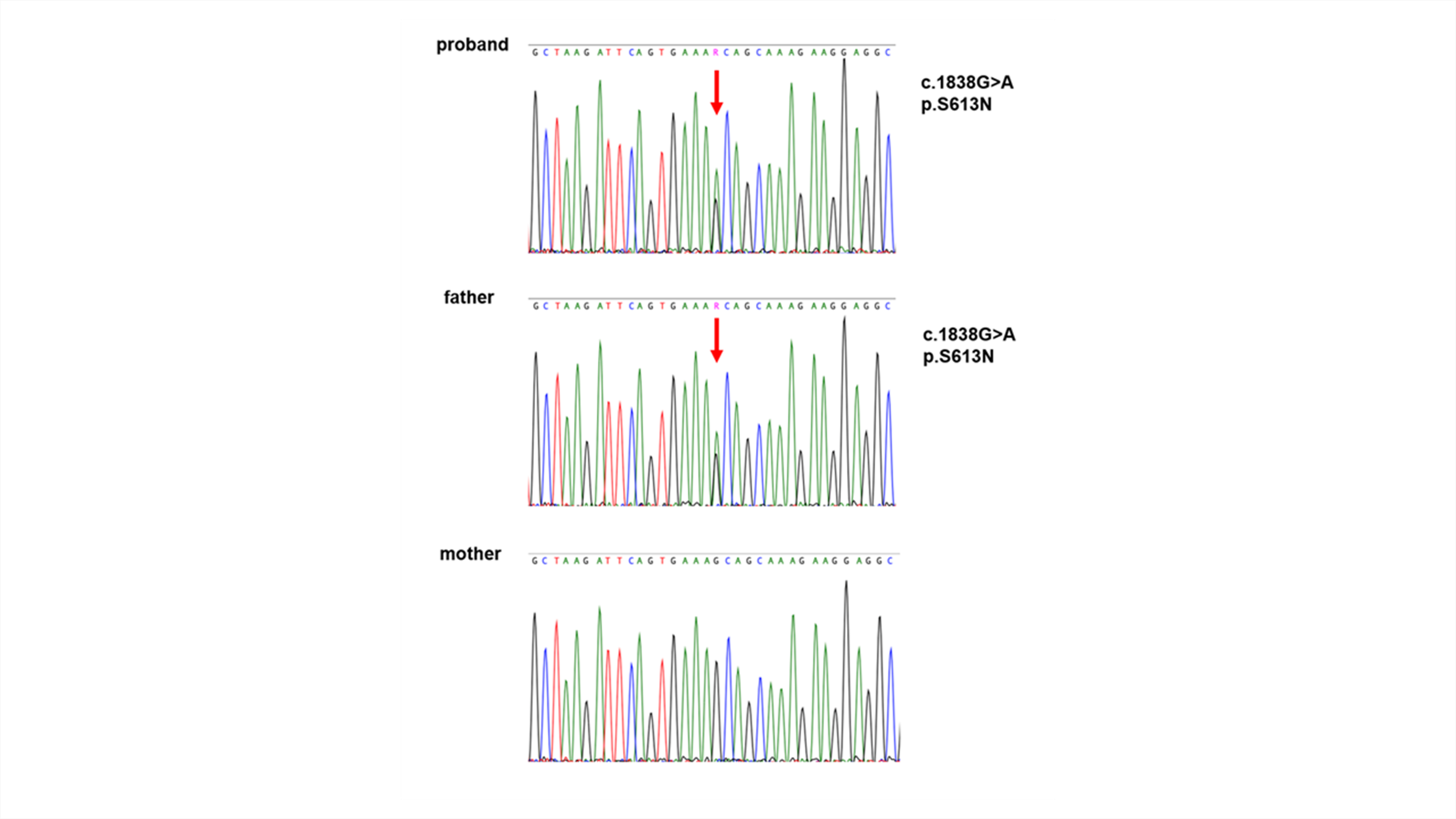

Supplement: Supplementary file 2 — High Resolution Image (TIF 11.8 MB) [file 10875_2025_1942_MOESM1_ESM.tif]

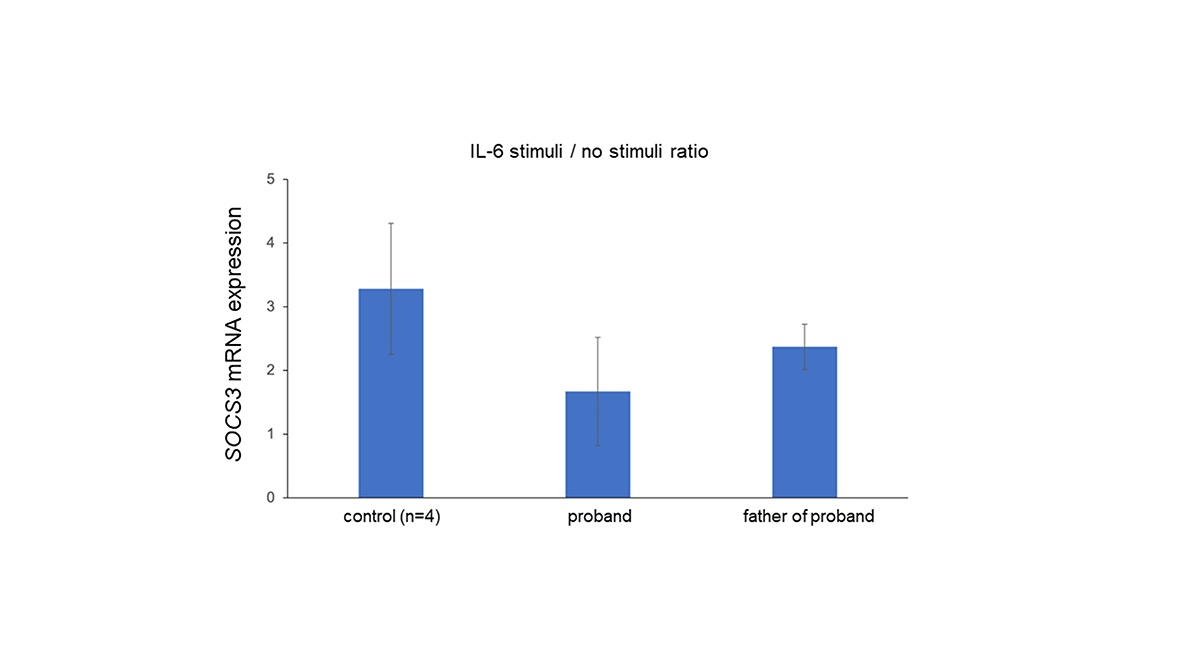

Supplement: Supplementary file 3 — PNG 29.6 KB [file 10875_2025_1942_Fig3_ESM.png]

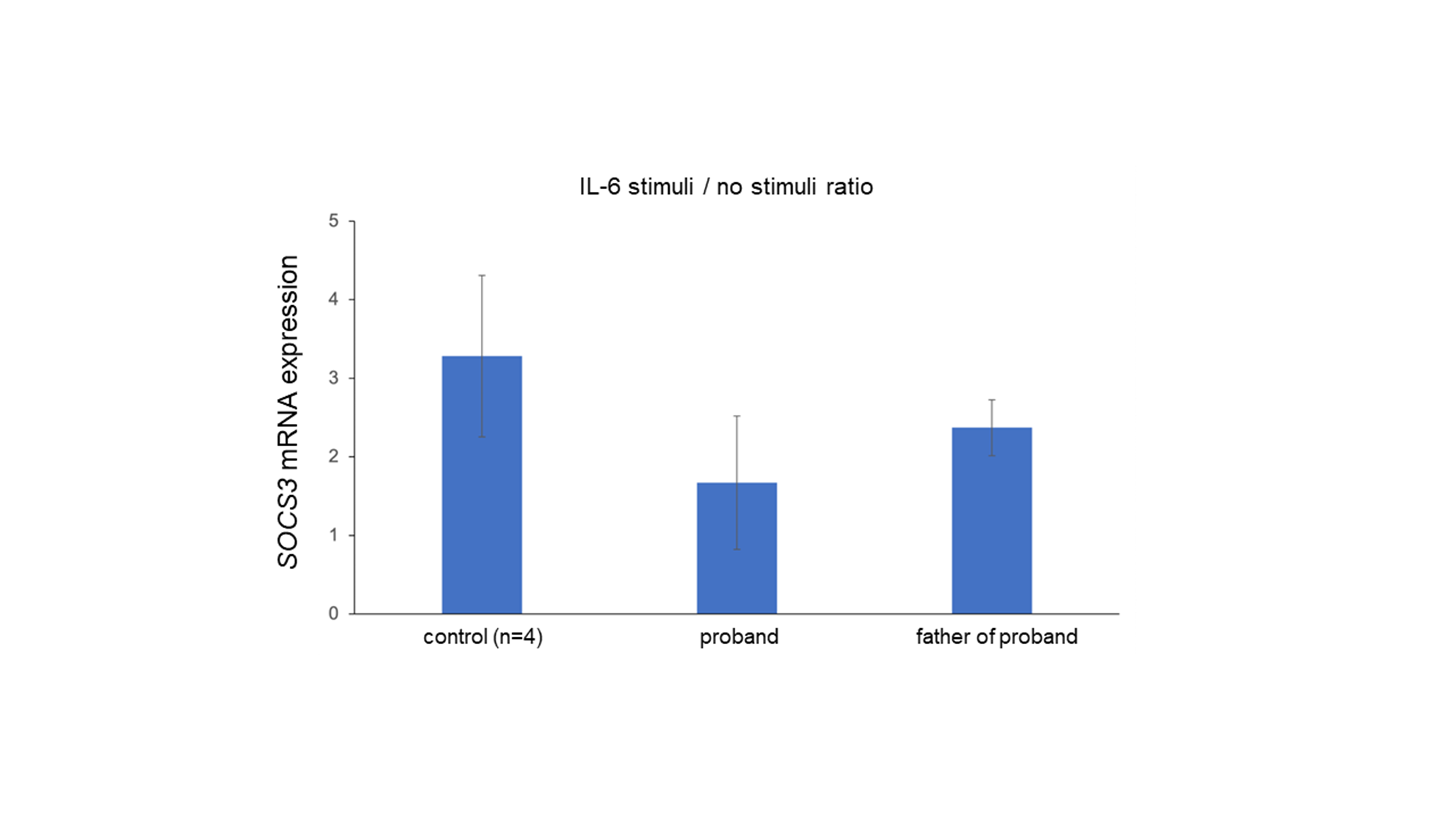

Supplement: Supplementary file 4 — High Resolution Image (TIF 1.13 MB) [file 10875_2025_1942_MOESM2_ESM.tif]

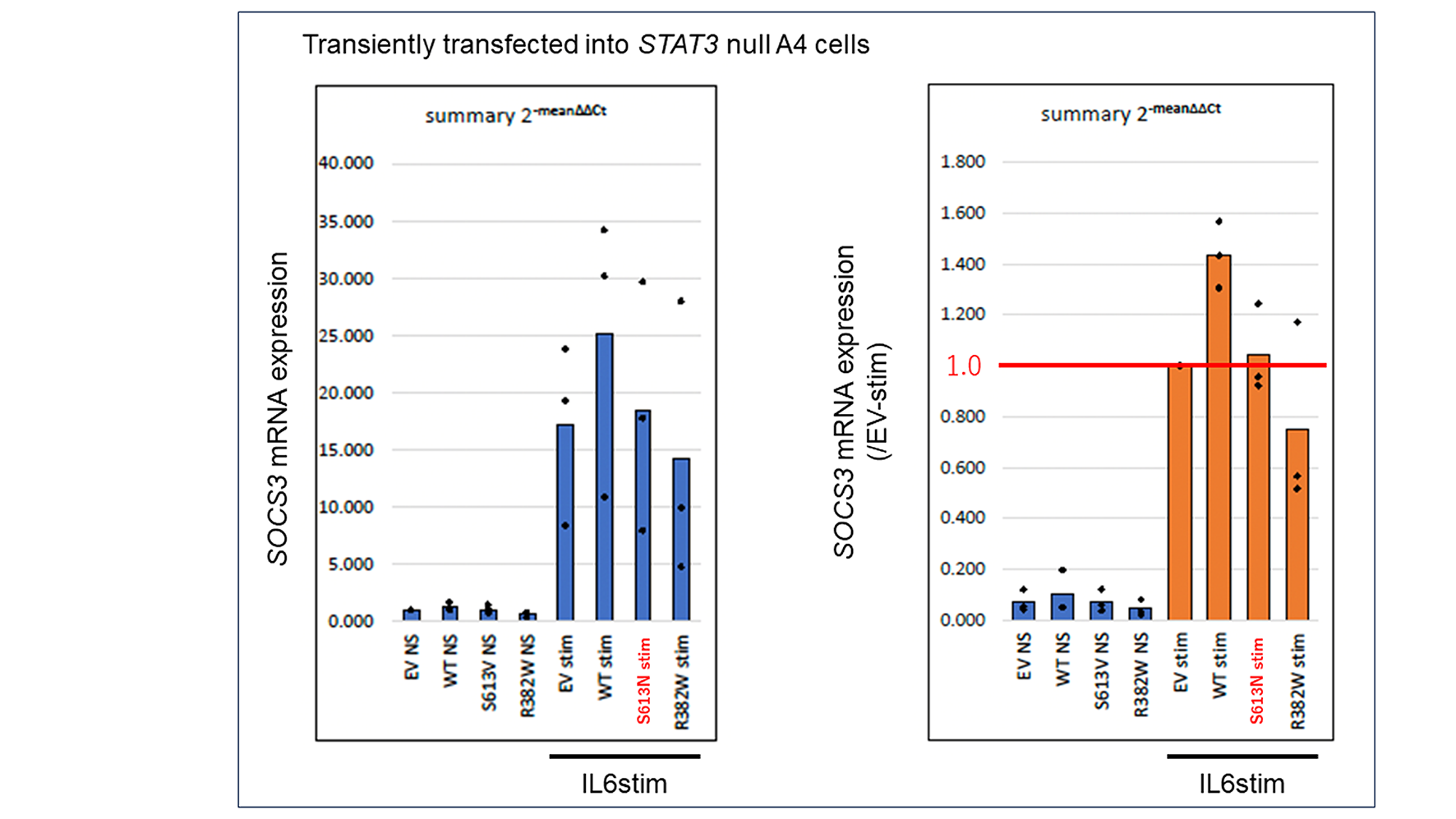

Supplement: Supplementary file 5 — PNG 356 KB [file 10875_2025_1942_Fig4_ESM.png]

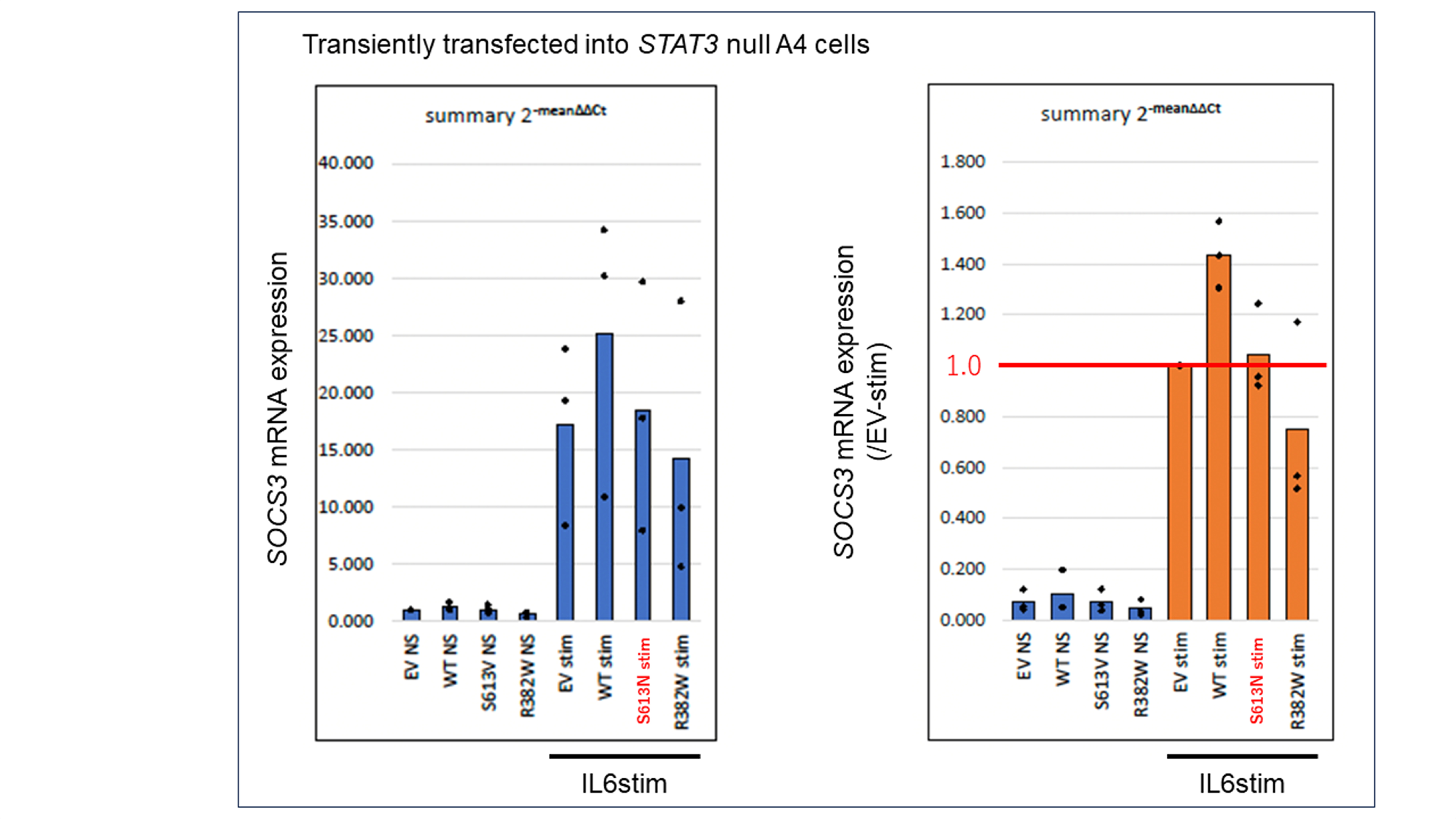

Supplement: Supplementary file 6 — High Resolution Image (TIF 12.4 MB) [file 10875_2025_1942_MOESM3_ESM.tif]
