# Supplementary material for: A Novel Hypomorphic STAT3 Gene Variant in a 7-year-old Male with Hyper-IgE Syndrome
Source: J Clin Immunol. 2025 Oct 20;45(1):146. doi: 10.1007/s10875-025-01942-7 (PMC12537600; doi:10.1007/s10875-025-01942-7)
Supplement: Supplementary file 7 — Supplementary file4 (DOCX 16.4 KB) [file 10875_2025_1942_MOESM4_ESM.docx]

**Supplementary Figure Legends (Revised)**

**Supplementary Figure 1.**
Sequences of *STAT3* from the patient and parents were verified using the Sanger method. A substitution (*c.1838G>A*) was found in the patient and father, but not in the mother.

**Supplementary Figure 2.**
mRNA expression levels of *SOCS3* in peripheral blood mononuclear cells measured with quantitative real-time PCR. Data are shown as the ratio (IL-6 30 ng/mL stimulation for 24 h / no stimulation) with mean ± SD for three independent experiments. No significant difference in IL-6–induced *SOCS3* expression levels was observed between control subjects (n = 4) and the patients.

**Supplementary Figure 3.**
mRNA expression levels of *SOCS3* in *STAT3^−/−^* A4 cells transiently transfected with EV, WT, or *STAT3* variants (S613N and R382W), measured with quantitative real-time PCR. After 24 h, cells were stimulated with or without IL-6 (10 ng/mL) for 45 min at 37 °C, and total RNA was extracted. *SOCS3* mRNA expression with the S613N variant was significantly decreased compared with WT (p < 0.05). The experiment was independently repeated three times.
